# Structural, Biochemical, and Phylogenetic Analysis of Bacterial and Fungal Carbohydrate Esterase Family 15 Glucuronoyl Esterases in the Rumen

**DOI:** 10.1007/s10930-024-10221-0

**Published:** 2024-08-17

**Authors:** Robert J. Gruninger, Maya Kevorkova, Kristin E. Low, Darryl R. Jones, Liam Worrall, Tim A. McAllister, D. Wade Abbott

**Affiliations:** 1 Lethbridge Research and Development Centre, Agriculture and Agri-Food Canada, Lethbridge, AB Canada; 2 Life Sciences Institute, University of British Columbia, Vancouver, BC Canada

**Keywords:** Glucuronoyl esterase, CAZyme, Carbohydrate esterase family 15, Neocallimastigomycota, Rumen

## Abstract

Glucuronoyl esterases (GEs) are carbohydrate active enzymes in carbohydrate esterase family 15 which are involved in the hydrolysis of lignin-carbohydrate complexes. They are encoded by a wide range of aerobic and anaerobic fungi and bacteria inhabiting diverse environments. The rumen microbiome is a complex microbial community with a wide array of enzymes that specialize in deconstructing plant cell wall carbohydrates. Enzymes from the rumen tend to show low similarity to homologues found in other environments, making the rumen microbiome a promising source for the discovery of novel enzymes. Using a combination of phylogenetic and structural analysis, we investigated the structure-function relationship of GEs from the rumen bacteria *Fibrobacter succinogenes* and *Ruminococcus flavefaciens*, and from the rumen fungus, *Piromyces rhizinflata*. All adopt a canonical α/β hydrolase fold and possess a structurally conserved Ser-His-Glu/Asp catalytic triad. Structural variations in the enzymes are localized to loops surrounding the active site. Analysis of the active site structures in these enzymes emphasized the importance of structural plasticity in GEs with non-canonical active site conformations. We hypothesize that interkingdom HGT events may have contributed to the diversity of GEs in the rumen, and this is demonstrated by the phylogenetic and structural similarity observed between rumen bacterial and fungal GEs. This study advances our understanding of the structure-function relationship in glucuronoyl esterases and illuminates the evolutionary dynamics that contribute to enzyme diversity in the rumen microbiome.

**Supplementary Information:**

The online version contains supplementary material available at 10.1007/s10930-024-10221-0.

## Introduction

Ruminants first evolved an estimated 50 million years ago (Mya) and are the most widely adapted livestock on earth, with ~ 200 species inhabiting environments ranging from the high arctic to the tropics [1]. The rumen is a large anaerobic chamber in the foregut of ruminants and is the principal site of feed digestion. The efficiency with which ruminants can utilize fibrous crop residues and by-products to produce milk and meat is unique amongst livestock, enabling the inclusion of plant materials in their diets that are unsuitable for human consumption. Like all mammalian herbivores, ruminants do not produce cellulolytic or hemi-cellulolytic enzymes to degrade ingested plant material; relying instead on a symbiotic association with rumen bacteria, fungi, methanogens, and protozoa to produce a diverse range of enzymes that break down the lignocellulose matrix in plant cell walls [2–4]. The enzymes produced by rumen microbes convert plant polysaccharides into sugars which are subsequently fermented to the volatile fatty acids (VFAs): acetate, propionate, and butyrate [2, 3]. The resulting VFAs and microbial protein are utilized by the animal for maintenance energy, growth, and lactation [5].

Plant biomass makes up the largest component of the feed consumed by ruminant livestock [6]. The lignocellulose matrix of plant cell walls is composed of a complex network of cellulose, hemicellulose, pectin, and the phenolic polymer lignin. The diversity of chemical groups and linkages in plant cell walls, and the extensive network of covalent linkages with lignin, make lignocellulose highly recalcitrant [7]. Substantial effort has been made to understand the role that carbohydrate active enzymes (CAZymes) play in breaking down the plant cell wall, however, the mechanisms employed by microbes to break down lignin-carbohydrate complexes (LCCs) are less well defined [8]. Microbes in aerobic environments express lignin-modifying enzymes including laccases, manganese peroxidases, and lignin peroxidases, which together with carbohydrate esterases, digest LCCs [9, 10]. The hydrolysis of LCCs in the gut is primarily attributed to the action of carbohydrate esterases [8, 11], and while it is generally thought that lignin digestion does not occur in anaerobic environments, recent evidence is challenging this dogmatic view [12].

Glucuronoyl esterases (GEs) within the carbohydrate esterase family 15 (CE15) esterase family, in a range of aerobic and anaerobic fungi and bacteria, have been identified as playing an important role in breaking down LCCs [8, 13, 14] by cleaving the ester bond between 4-*O*-methyl-d-glucuronyl in xylan and alcohol groups in lignin [15, 16]. There is mounting evidence that the removal of glucuronic acid residues significantly improves the saccharification of lignocellulosic feed stocks [17–19], suggesting that the activity of these enzymes acts in synergy with glycosyl hydrolases. As of Aug 10 2023, of the ~ 747 unique carbohydrate esterase family 15 esterases identified in the CAZy database, only 22 have been biochemically characterized and have generally been found to exhibit narrow substrate specificity, with little to no activity against the model substrates typically hydrolyzed by esterases [15, 20]. The structures of 9 GEs have been determined from the carbohydrate esterase family 15 esterase family and all share a conserved α/β-hydrolase fold. Outside of the core scaffold, GEs display structural variability in the loops surrounding the catalytic site, and this hypothesized to contribute to their in-vivo specificity against LCCs [8]. Bacterial GEs typically possess variable insertions in an N-terminal loop (RegN) and at 3 regions surrounding the active site referred to as Reg1-Reg3. These insertions make bacterial GE active sites deeper, and more contoured than the active sites observed in fungal counterparts [15, 16, 21]. GEs share an invariant Ser-His-Glu/Asp catalytic triad and employ a conserved catalytic mechanism that involves the formation of an acyl-enzyme covalent intermediate, which is subsequently cleaved through acid-base hydrolysis. Both the acylation and deacylation steps involve the formation of a tetrahedral transition state and are stabilized by the catalytic histidine and a conserved arginine residue that is found adjacent to the catalytic serine [22, 23]. The position of the catalytic nucleophile and general base is highly conserved; however, the general acid has been observed at two distinct locations in the structure [24]. To reflect this variability, GEs are classified as possessing a canonical (CE15-B) or non-canonical (CE15-A) catalytic triad. This classification is based on the structural arrangement of the catalytic acid and whether they are located on loops following β7 or β8, respectively. It is unclear how these differences in the arrangement of the catalytic triad impact enzyme function, but it may be related to differences in the structure of the natural substrate targeted by the enzymes [24]. Catalytic residues of classical α/β-hydrolase serine esterases have also been classified as having a canonical (A-type) and non-canonical (B-type) arrangement based on the position of the general acid and whether they are located on a loop following β7 or β6, respectively [23–25]. Unfortunately, the discrepancy between GE and classical α/β-hydrolase nomenclature can cause confusion. We note that we are considering the spatial arrangement of catalytic residues using the GE specific classification scheme put forth by Ernst and colleagues [24].

The microbial diversity of the rumen represents a vast genetic pool that is ideal for the discovery of novel enzymes targeting plant cell wall carbohydrates. Many of the enzymes encoded by rumen microbes have low homology to related proteins found in other environments, which makes the rumen microbiome a valuable resource for the discovery of novel biocatalysts [26]. To this end, we have conducted an analysis of GE diversity within rumen microbes and examined the structure-function relationship of three GEs from *Fibrobacter succinogenes*, *Ruminococcus flavefaciens*, and *Piromyces rhizinflata*. This work aimed to contribute additional knowledge about the diversity of GEs within the carbohydrate esterase family 15 esterase family and to expand our understanding of the structure-function relationship within these enzymes.

## Methods

*Phylogenetic analysis of the carbohydrate esterase family 15 family of esterases*: A phylogenetic tree of the carbohydrate esterase family 15 family of esterases was built using SACCHARIS on August 10 2023 [27]. GEs from rumen bacteria and fungi were identified within the Hungate 1000 dataset [28] and the genomes of Neocallimastigomycota that are available in Mycocosm [29]. The carbohydrate esterase family 15 HMM model was obtained from dbCAN2 [30], and HMMER3 [31] was used to search the rumen microbe genome dataset. Carbohydrate esterase family 15 sequences in the CAZy database (http://www.cazy.org/; [32, 33]) were downloaded (August 10 2023), combined with the rumen carbohydrate esterase family 15 esterase dataset, and dereplicated using CD-HIT [34, 35] using a sequence identity cut-off of 100% to remove duplicate sequences. The resulting dereplicated sequences were used as input to SACCHARIS for phylogenetic analyses [27] (https://github.com/saccharis/SACCHARIS_2). Briefly, CD-HIT parsed sequences were pruned to the carbohydrate esterase family 15 domain using dbCAN2 [30] and then aligned using MUSCLE [36]. ModelTest-NG [37] was used for best-fit model selection using the sequence alignment, and FastTree [38] was used to generate the phylogenetic tree.

*Protein production and purification*: Genes encoding GEs from *Ruminococcus flavefaciens* (the C-terminal carbohydrate esterase family 15 esterase domain of CesA herein referred to as *Rf*CE15), *Fibrobacter succinogenes* (*Fs*CE15), and the rumen fungus *Piromyces rhizinflata* (*Pr*CE15) were synthesized and codon optimized for protein production in *Escherichia coli* (Thermo Fisher). The Gateway cloning system was used to generate both C- and N-terminal His_6_ tagged destination vectors from the Champion™ pET300/NT-DEST and pET301/CT-DEST Gateway™ Vector Kit (Thermo Fisher). Protein production constructs were sequence verified and transformed into BL21(DE3) *E. coli* (New England Biolabs) for protein production. Cells were grown in Lysogeny Broth (LB) supplemented with 100 µL mL^-1^ ampicillin to an optical density (600 nm) of 0.6–0.8 at which point protein production was induced with the addition of Isopropyl β-d-1-thiogalactopyranoside (IPTG) to a final concentration of 1 mM. Protein production was carried out at 25 °C for 18 h in an incubated shaker. Cells were harvested by centrifugation at 4,000 × g for 20 min at 4 °C. The resulting cell pellet was resuspended in 20 mL of lysis buffer (0.5 M NaCl and 20 mM HEPES pH 7.5) and a protease inhibitor tablet (Roche) was dissolved in the cell suspension. Cells were ruptured by sonication and cell debris was removed by centrifugation at 30,000 × g for 45 min at 4 °C. Protein was purified to homogeneity via a combination of Ni-IMAC (nickel immobilized metal-affinity chromatography) and size exclusion chromatography with a Superdex 200 column. Protein concentration was determined by measuring absorbance at 280 nm using the molar absorption coefficient calculated by ProtParam [39]. Purified protein was stored in 20 mM HEPES (pH 7.5) and 200 mM NaCl. Protein was used immediately, or flash frozen in liquid nitrogen and stored at -80 °C.

*Protein crystallization*: Initial crystallization trials were carried out with Crystal Screen 1 + 2 (Hampton Research) and PACT premier™/JCSG-plus™ (Molecular Dimensions) screens in Intelli-Plate 96 − 2 sitting drop crystallization plates (Hampton Research). Protein was mixed in a 1:1 ratio of protein: reservoir with a final drop volume of 2 µl. 100 µL of crystallization solution was added to reservoirs. Crystallization hits were optimized in a 24 well sitting drop Cryschem crystallization plate (Hampton Research). Diffraction quality crystals were obtained for the C-terminally His-tagged construct of *Fs*CE15 and *Rf*CE15 and the N-terminally His-tagged construct of *Pr*CE15. Optimized crystallization conditions were: 25 mg/ml *Fs*CE15, 10 mM ZnCl_2_, 18% PEG 6000, and 100 mM sodium acetate-acetic acid buffer (pH 5.0); 30 mg/ml *Rf*CE15, 11-13% 2-propanol, 18-20% PEG 4000; 30 mg/ml *Pr*CE15, 100 mM HEPES (pH 7.0), 20% PEG 6000. Crystals were mounted in nylon loops in the presence of 18% glycerol as a cryoprotectant and flash frozen in liquid nitrogen.

*Data collection and structure determination*: Diffraction data was collected at -173 °C on beamline 08ID-1 at the Canadian Light Source (Saskatoon, SK) at a wavelength of 0.97949 Å. Data was integrated with XDS [40] and scaled with Scala and the CCP4 suite of programs [41]. The structure was solved by molecular replacement with Phaser-MR in Phenix [42]. The structure of the GE from *Trichoderma reesei*, Cip2 (PDB: 3PIC, [43]) was trimmed to a poly-ala backbone and used as a search model to solve the structures of *Pr*CE15 and *Fs*CE15. Using a similar approach, the structure of *Rf*CE15 was solved using the GE from *Sporotrichum thermophile* (PDB: 4G4G, [44]) as a search model. The resulting models served as a starting point for iterative cycles of TLS (Translation–Libration–Screw-rotation), positional, real space and *B*-factor refinement in Phenix [42], followed by manual model building with Coot [45]. Refinement was monitored by flagging 5% of all reflections as “free” [46]. Stereochemistry of the model was monitored throughout model building and refinement using the Phenix Validation tools. Statistics for data collection and refinement are shown in Table 1.

*Enzyme Assays: *d-glucuronic acid methyl ester (MeGlcA), d-galacturonic acid methyl ester (MeGalA), Benzyl d-glucuronate (BnzGlcA), and Allyl D-glucuronate (AllylGlcA) (all from Carbosynth) were dissolved in 99% DMSO and filtered with a 0.45 μm syringe filter. A 3-component buffer system (25 mM acetic acid, 25 mM MES, 50 mM Tris-HCl, pH variable) was used for all assays as previously described [16]. Uronic acid formation was measured continuously in a BioTec Synergy™ HT multi-detection microplate reader using the d-Glucuronic Acid/d-Galacturonic Acid (K-Uronic) Assay Kit (Megazyme). Due to substrate instability, a control that did not contain enzyme served as an auto-hydrolysis blank. The blank reaction was measured continuously in parallel with the enzyme containing reactions, and the rate of auto-hydrolysis was subtracted from enzyme catalyzed hydrolysis. Assays to examine the effect of pH on enzyme activity were conducted using BnzGlcA at a concentration of 2 mM and varying the pH of the 3-component buffer. Kinetic assays were performed with BnzGlcA (0.5–10 mM) at pH 7.0 and kinetic parameters were calculated using SigmaPlot. To examine substrate specificity, the specific activity of the enzymes against 2 mM MeGlcA, MeGalA, and AllylGlcA was measured at pH 7.0 and the activity against these substrates was compared relative to BnzGlcA and expressed as a percentage of 100%.

*Thermal stability of enzymes*: A Protein Thermal Shift™ qPCR assay (Thermo Fisher) was performed in a qPCR plate MicroAmp™ Fast Optical 96-Well Reaction Plate, using a Quantstudio 6 Flex qPCR system (Applied Biosystems). Stability assays were carried out in 20 µL reactions containing: 2 µL 200 X diluted SYPRO™ Orange Protein Gel Stain (Thermo Fisher), 1 µL 20 X three component buffer pH 7, 15 µL of 14 µM enzyme (C_f_ = 10.5 µM) (replaced with d^2^H_2_O for blanks), and 2 µL of additive(s). Additives in the assay were d_2_H_2_O (Control), 10 mM Tris(2-carboxyethyl)phosphine hydrochloride (TCEP), 100 mM and 500 mM NaCl. Four technical replicates were included for each unique condition examined in the assay and the experiment was repeated twice. The T_m_ was calculated using the program TSA CRAFT [47].

## Results

**Fig. 1:**
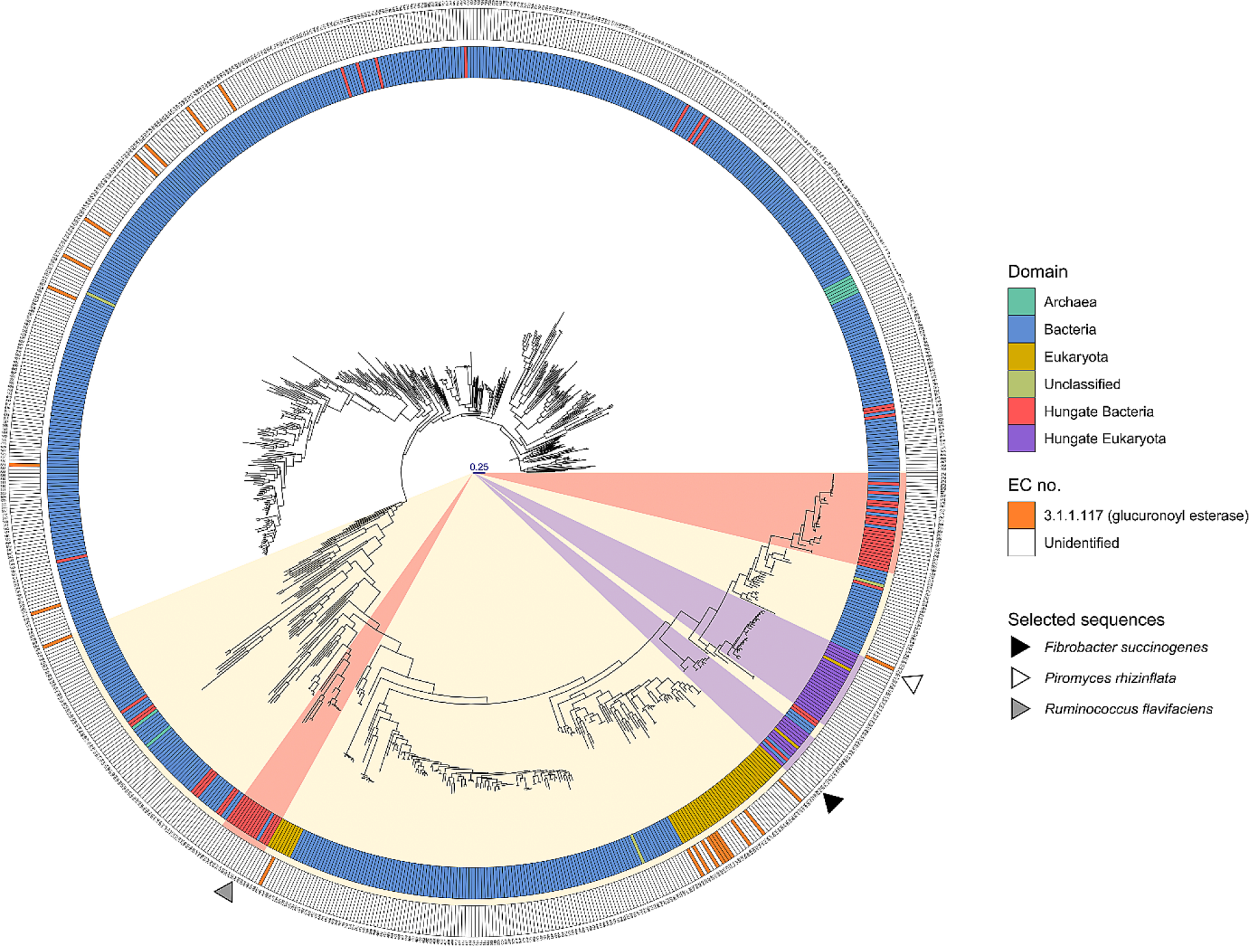
Phylogenetic analysis of rumen carbohydrate esterase family 15 esterases. Glucuronoyl esterases in the carbohydrate esterase family 15 esterase family from the CAZy database (accessed August 10, 2023) and the rumen collection were clustered by their sequence identity, used as query sequence inputs for SACCHARIS [27], and embedded into a mid-point rooted phylogenetic tree. Coloured rings surrounding the tree highlight sequence characteristics by domain (inner) and EC number and CAZy database annotated function (outer). Carbohydrate esterase family 15 esterases from anaerobic gut fungi and rumen bacteria are highlighted in purple (inner ring) and red, respectively. Proteins that have been biochemically characterized are shown in orange (outer ring). Proteins characterized in this study are highlighted with a triangle on the outside of the circle. Sequences used to generate the tree are numbered and accession codes provided in Supplementary Data 1

*Phylogenetic analysis of carbohydrate esterase family 15 esterases in the rumen:* A search of the Hungate 1000 collection identified 65 carbohydrate esterase family 15 esterases encoded in 53 of the 418 bacterial genomes. The majority (45/53) of the rumen bacteria which encoded a GE tended to possess a single GE. Multiple copies of carbohydrate esterase family 15 esterases were primarily identified in bacteria within the phylum Bacteroidota (*Bacteroides* sp, Porphyromonadaceae, and *Prevotella*). An unknown Lachnospiraceae was the only bacteria outside of the Bacteroidota with multiple carbohydrate esterase family 15 esterase containing ORFs. A search of the 10 genome sequences of anaerobic gut fungi (AGF) within the phylum Neocallimastigomycota identified an additional 36 carbohydrate esterase family 15 esterases. All AGF genomes were found to encode multiple genes for GEs, ranging in number from 2 to 6 orthologues, with an average of 4 per genome (Supplementary Data 1). In agreement with previous phylogenetic analysis of the carbohydrate esterase family 15 family, we observed that the proteins formed 2 super clades (Fig. 1). Clade 1 consisted mainly of bacterial carbohydrate esterase family 15 esterases, with a small cluster of archaeal sequences, while clade 2 (highlighted in light yellow) consisted of bacterial, archaeal, and fungal enzymes. Despite the identification of carbohydrate esterase family 15 esterases in archaea, they were notably scarce (8 out of 826), all of which originated from a family of extreme halophiles within the order Halobacteriales. The rumen carbohydrate esterase family 15 esterases were found predominantly in 4 main clusters within clade 2 (Fig. 1; rumen eukaryotic clusters highlighted in purple, rumen bacterial clusters highlighted in red).

*Structure of rumen GEs*: Although we attempted to express genes and crystallize proteins of GEs from a range of rumen microbes, only *Fs*CE15, *Pr*CE15, and *Rf*CE15 produced diffraction quality crystals. Statistics for the data collection and refinement are shown in Table 1. Crystals of *Fs*CE15 belonged to space group P4_1_2_1_2 with a single protein molecule in the asymmetric unit. The structure of *Fs*CE15 was refined to R_work_/R_free_ of 22.8%/28.5% (respectively) using data to a maximum resolution of 1.93 Å. Crystals of *Pr*CE15 belonged to space group P6_1_ with a single protein molecule in the asymmetric unit. The structure of *Pr*CE15 was refined to R_work_/R_free_ of 22.9%/26.9% using data with a maximum resolution of 2.54 Å. Crystals of *Rf*CE15 belonged to space group P2_1_2_1_2_1_ with 2 protein molecules in the asymmetric unit. The structure of RfCE15 was refined to R_work_/R_free_ of 16.8%/19.0% using data with a maximum resolution of 1.25 Å. All of the residues in the three models were found in allowable regions of the Ramachandran plot. Structures have been deposited to the protein databank with accession codes: 8TRU (*Fs*CE15), 8TRX (*Pr*CE15), 8TSE (*Rf*CE15).

**Table 1:** X-ray data collection and refinement statistics for the structures of the glucuronoyl esterases from *F. succinogenes* (*Fs*CE15), *P. rhizinflata* (*Pr*CE15), and *R. flavefaciens* (*Rf*CE15). Statistics for the highest-resolution shell are shown in parentheses

Data Collection	*Fs*CE15	*Pr*CE15	*Rf*CE15
Beamline	CLSI 08ID-1	CLSI 08ID-1	CLSI 08ID-1
Wavelength (Å)	1.0334	1.0334	1.0334
Space group	P 4_1_ 2_1_ 2	P 6_1_	P 2_1_ 2_1_ 2_1_
Cell Dimensions			
*a*,* b*,* c* (Å)	57.28 57.28 222.16	139.68 139.68 125.83	51.95 60.87 124.99
α, β, γ	90.00, 90.00, 90.00	90.00, 90.00, 120.00	90.00, 90.00, 90.00
Resolution (Å)	45.31–1.93 (1.99–1.93)	46.74–2.54 (2.63–2.54)	43.6–1.25 (1.30–1.25)
R_merge_	0.167 (1.02)	0.0813 (0.933)	0.0882 (1.05)
R_pim_	0.0494 (0.291)	0.0265 (0.300)	0.0358 (0.427)
CC_1/2_	0.998 (0.8940)	0.999 (0.858)	0.999 (0.724)
Wilson *B*-factor	26.4	59.7	12.8
< I/σI>	10.99 (2.86)	19.03 (2.76)	11.27 (1.85)
Completeness (%)	99.97 (100.0)	99.96 (100.0)	99.96 (100.0)
Redundancy	12.5 (13.2)	10.4 (10.6)	7.0 (6.9)
Total reflections	362,382 (36,927)	478,327 (48,424)	773,121 (74,487)
Unique reflections	28,949 (2791)	45,952 (4555)	110,196 (10,867)
***Refinement***			
Resolution (Å)	1.93	2.54	1.25
R_work_/R_free_	0.228/0.285	0.229/0.269	0.168/0.190
Protein residues	380	773	409
*Non-hydrogen atoms*			
Protein	2858	5835	3222
Ligands	0	0	12 (GOL)
Water	108	83	335
*RMSD*			
Bond lengths (Å)	0.007	0.01	0.005
Bond angles (°)	0.84	1.08	0.81
*Ramachandran* (%)			
Preferred	95.99	92.85	96.56
Allowed	4.01	7.15	3.44
Disallowed	0	0	0
*B-factors* (Å^2^)			
Protein	33.77	62.99	14.89
Ligands	0	0	20.91 (GOL)
Water	32.1	54.59	23.04
PDB ID	8TRU	8TRX	8TSE

**Fig. 2:**
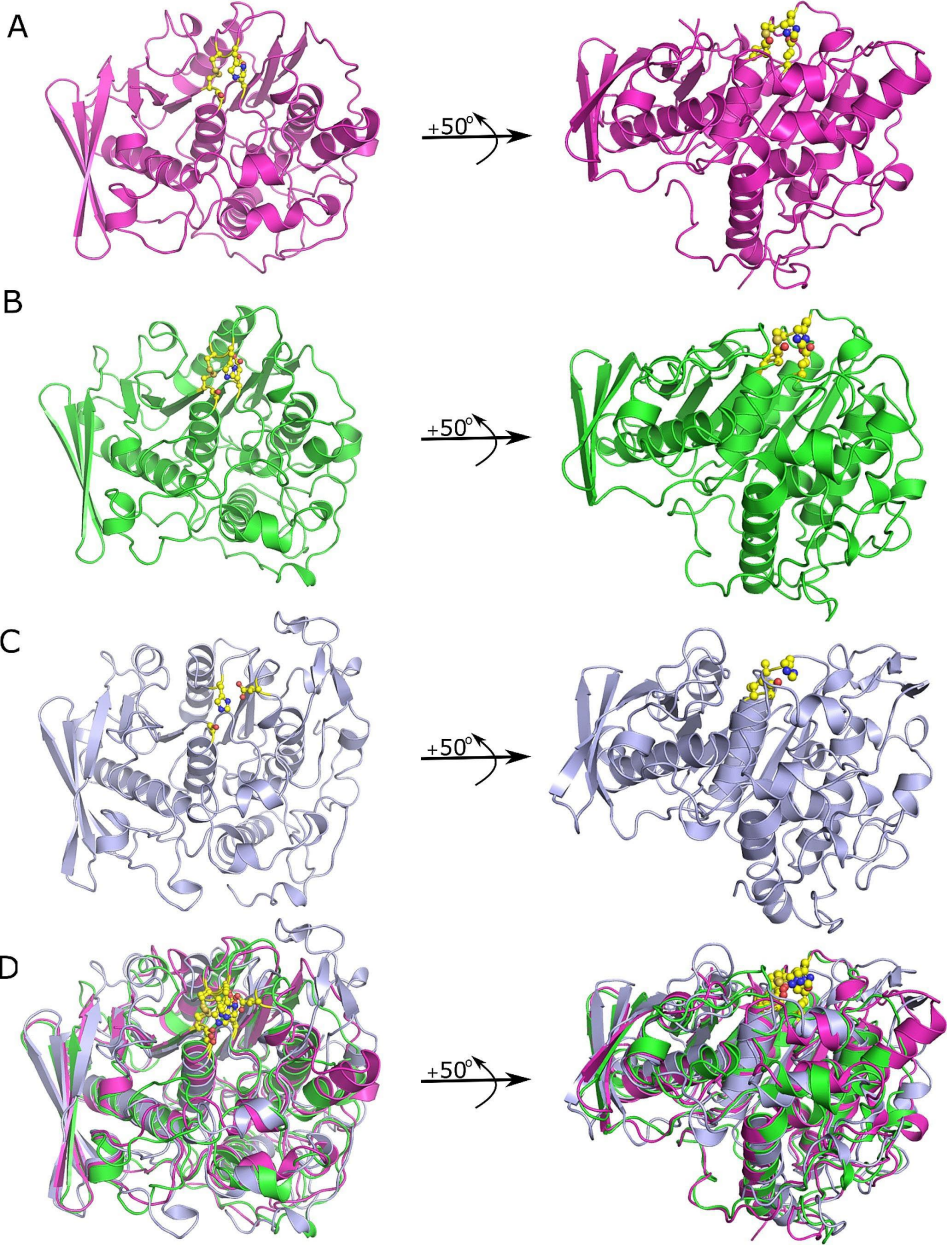
Comparison of the fold of rumen glucuronoyl esterases within the carbohydrate esterase family 15 esterase family. (**A**) *Fibrobacter succinogenes* (*Fs*CE15), (**B**) *Piromyces rhizinflata* (*Pr*CE15), and (**C**) *Ruminococcus flavefaciens* (*Rf*CE15) adopt the canonical α/β-hydrolase fold that is seen in all of the GE structures solved to date. (**D**) Structure-based least squares structural superposition of *Fs*CE15, *Pr*CE15 and *Rf*CE15. Catalytic residues, and the conserved disulfide bridge in the active sites of FsCE15 and PrCE15 are shown as yellow ball and sticks. Figures were generated with PyMol

*Rumen GEs adopt a canonical α/β hydrolase fold: Fs*CE15, *Pr*CE15, and *Rf*CE15 adopted the canonical α/β-hydrolase fold that is seen in all of the GE structures solved to date (Fig. 2). The fold of all three enzymes consists of a central twisted 10 stranded mixed β-sheet flanked by α-helices on either side. Structure-based least square superpositions of *Pr*CE15, *Fs*CE15, and *Rf*CE15 had Cα root mean square deviations (RMSD) of < 1.3 Å with the structural variation being limited to surface exposed loops (Fig. 2D). All of the enzymes lacked insertions at the RegN and Reg1-Reg3 variable regions that are typically observed in bacterial GEs and displayed a flat active site cleft common with fungal GEs.

**Fig. 3:**
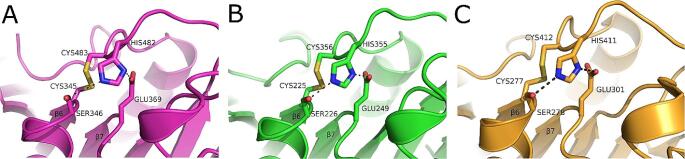
Canonical configuration of catalytic residues in the active site of glucuronoyl esterases within the CE15-B subfamily. (**A**) Glucuronoyl esterase from *Fibrobacter succinogenes*, *Fs*CE15, (**B**) Glucuronoyl esterase from *Piromyces rhizinflata*, *Pr*CE15, (**C**) Glucuronoyl esterase from *Trichoderma reesei*, Cip2. All figures were generated with PyMOL

*Comparative Analysis of the FsCE15 and PrCE15 Active Sites:* To identify the putative catalytic residues in *Fs*CE15 and *Pr*CE15, a structure based superposition with the *T. reesei* GE Cip2 was generated. *Fs*CE15 and *Pr*CE15 had an RMSD of 0.87 Å and 1.1 Å with Cip2, respectively. The location and identity of the experimentally validated catalytic residues of Cip2 were conserved in both *Fs*CE15 and *Pr*CE15. The putative catalytic nucleophile, general base, and general acid of *Fs*CE15 are Ser346, His482, and Glu369, respectively (Fig. 3A). A similar examination of these resides in *Pr*CE15 putatively identifies the catalytic residues as Ser226, His355, and Glu249 (Fig. 3B). The catalytic residues of *Fs*CE15 and *Pr*CE15 are positioned in the canonical conformation characteristic of CE15-B subfamily [24] with the general acid located on a loop at the end of β7 (Fig. 3). *Fs*CE15 and *Pr*CE15 have a conserved disulfide bond between residues Cys345 - Cys483 and Cys225 - Cys356, respectively, which anchors the general base within optimal hydrogen bonding distance (2.7 Å) to the catalytic serine. This disulfide is conserved amongst CE15-B subfamily GEs that adopt a canonical catalytic triad and can be seen in the glucuronoyl esterase Cip2 from (Fig. 3C), *Lentithecium fluviatile Lf*CE15C [48], *Cerrena unicolor Cu*GE [24], and *S. thermophile St*CE15 [44]. The glycine and lysine residues of the nucleophilic elbow GXSRXGK motif [48] are conserved in both *Fs*CE15 and *Pr*CE15. The arginine following the catalytic serine in the motif contributes to the stabilization of the transition state, and while the arginine is conserved in *Fs*CE15 it is replaced with a tyrosine in *Pr*CE15. Interestingly, it was more common for this residue to be tyrosine in AGF GEs (26 out of 35 sequences) with arginine found at this position in the nine remaining rumen enzymes.

**Fig. 4:**
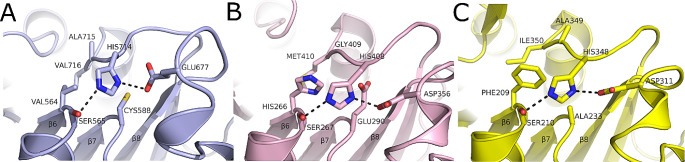
Non-canonical configuration of catalytic residues in the active site of glucuronoyl esterases within the CE15-A subfamily (**A**) C-terminal glucuronoyl esterase domain from *Ruminococcus flavefaciens* CesA, *Rf*CE15 (**B**) Glucuronoyl esterase from *Optitus terrae*,* Ot*CE15A and (**C**) Glucuronoyl esterase from *Caldicellulosiruptor kristjansonii*,* Ck*CE15A. All figures were generated with PyMOL

*RfCE15 catalytic residues adopt a non-canonical conformation:* Similar to *Fs*CE15 and *Pr*CE15, *Rf*CE15 adopts the same α/β-hydrolase fold that is highly conserved amongst GEs in the carbohydrate esterase family 15 CAZy family (Fig. 2D). *Rf*CE15 is the C-terminal GE domain of a multi-modular enzyme from *R. flavefaciens* called CesA. CesA was first described by Aurilia an colleagues as an acetylxylan esterase with a domain of unknown function (DUF) [47]. The DUF was later characterized as a GE belonging to the carbohydrate esterase family 15 CAZy family [48]. The catalytic residues of *Rf*CE15 are observed in a non-canonical configuration, with the putative general acid Glu677 located on a loop at the end of β8; making *Rf*CE15 a member of the CE15-A GE subfamily. The nucleophilic serine (Ser565) is positioned similarly to the conformation observed in all other carbohydrate esterase family 15 esterases and located at the base of the active site in the characteristic GXSRXGK sequence motif. The glycine, lysine, and arginine residues within this motif were all conserved in *Rf*CE15. The general base (His714) is located on a loop directly above the catalytic serine, but is shifted by 2.2 Å relative to the position of this loop in *Fs*CE15 and *Pr*CE15. The shift in this position accommodates the non-canonical conformation of the general acid residue (Fig. 4A). Despite the shift in Cα of His714, the imidazole ring occupies a similar location as that found in the other GEs, with His714 Nε2 making a 2.8 Å hydrogen bond with Oγ of Ser565, and a 2.9 Å bidentate hydrogen bond with the carboxyl functional group of Glu677 (Fig. 4A).

Structural differences in *Rf*CE15 were also observed in flexible loops located on the enzyme’s surface. Most notably, a 16 amino acid insertion from residues Gly603-Tyr619 forms an extended Ω-loop that wraps around the backside of the general base loop. Despite the presence of this large insertion, the surface profile of *Rf*CE15’s active site is a flat similar to that observed in previously characterized fungal GEs and in *Fs*CE15 and *Pr*CE15 (Fig. 2C). As observed in other CE15-A subfamily GEs, the conserved disulfide bond found in CE15-B GEs, was not observed in *Rf*CE15. Instead, these residues are Val564 and Val715 and form a hydrophobic pocket that is composed of an extended network of Van der Waals interactions between Ile114, Ile121, Met228, Phe314, ILE316, Ile366, and Met369. The contacts between the Ω-loop, the residues in the hydrophobic pocket, and the general base loop may function to stabilize the position of the general base in *Rf*CE15. Examining the equivalent region in other structurally characterized GEs in the CE15-A subfamily from *Optitus terrae* (PDB: 6gs0, *Ot*CE15A) (Fig. 4B) and *Caldicellulosiruptor kristjansonii* (PDB: 7nn3, *Ck*CE15A) (Fig. 4C) revealed that the residues in this pocket are not conserved. Despite the lack of sequence conservation, this region displays extensive non-covalent contacts with the general base loop, which may function to stabilize the general base loop, fulfilling a role similar to that of the disulfide bond in CE15-B GEs. Notably, *Ot*CE15A and *Ck*CE15A have Reg2 insertions typical of bacterial GEs, but that is not observed in *Rf*CE15, that also appears to contribute to stabilizing the general base loop in place of the conserved disulfide.

*Biochemical Characterization of FsCE15*,* PrCE15*,* and RfCE15*: The biochemical activity of *Fs*CE15, *Pr*CE15, and *Rf*CE15 was examined using the model substrates: MeGlcA, MeGalA, BnzGlcA, and AllylGlcA. All of the enzymes cleaved BnzGlcA with specific activities ranging from 2.66 U/min to 14.9 U/min (Table 2). The specific activity of *Pr*CE15 and *Rf*CE15 against BnzGlcA was approximately 5X higher than that observed with *Fs*CE15. None of the enzymes were active against MeGlcA or MeGalA. All of the enzymes cleaved AllylGlcA, however the activity was 85–90% lower than the activity observed with BnzGlcA. Based on the substrate preference of all three enzymes, all subsequent biochemical assays were conducted using BnzGlcA. The optimal pH for each enzyme was determined (Supplementary Fig. 1). *Fs*CE15 has a broad pH profile with maximal activity at pH 8.0. It retains ≥ 90% relative activity against BnzGlcA over a pH range from 6.5 to 8.5. *Rf*CE15 and *Pr*CE15 had optimal activity at slightly lower pH values, 6.5-7.0 and 7.0-7.5, respectively. We determined apparent steady state kinetic parameters for *Pr*CE15 and *Rf*CE15. Both enzymes displayed similar affinities for BnzGlcA; with *Pr*CE15 and *Rf*CE15 having apparent K_m_ values of 5.0 ± 0.4 mM and 6.0 ± 0.4 mM, respectively (Table 2). The apparent *k*_*cat*_ of *Pr*CE15 was determined to be 22.3 ± 0.7 s^-1^, compared to an apparent *k*_*cat*_ of 91.2 ± 0.8 s^-1^ for *Rf*CE15. Unfortunately, due to high background absorbance levels above 10 mM substrate, the reaction between *Fs*CE15 and BnzGlcA could not be adequately saturated and we were unable to determine steady-state kinetic parameters for this enzyme. It should be noted that substrate concentrations used to determine steady-state kinetic parameters should ideally reach 10x the concentration of the enzymes K_m_. Unfortunately, due to the low affinity that all three enzymes displayed for the synthetic substrate BnzGlcA, and the high background absorbance, it was not possible to reach saturating substrate concentrations in any of our assays. Given this limitation, the reported kinetic constants should be considered apparent values and interpreted cautiously. Consistent with previously characterized GEs, none of the enzymes had detectable activity against para-nitrophenyl linked substrates.

**Table 2:** Catalytic activity of *Fs*CE15, *Pr*CE15 and *Rf*CE15 against Benzyl D-glucuronate

Enzyme	pH optimum	Specific activity (µmol min^-1^mg^-1^)	Apparent K_m_ (mM)	Apparent k_cat_ (s^-1^)	Apparent k_cat_/ K_m_ (s^-1^M^-1^)
*Pr*CE15	7.5	12.5 ± 0.8	5.0 ± 0.4	22.3 ± 0.8	4485 ± 376
*Rf*CE15	6.5	14.9 ± 0.3	6.0 ± 0.4	29.7 ± 1.0	4950 ± 392
*Fs*CE15	8.0	2.66 ± 0.08	ND*, Reaction could not be saturated		

*Not determined

*Thermal stability of glucuronoyl esterases:* The thermal stability of *Fs*CE15, *Pr*CE15, and *Rf*CE15 was examined using a Thermofluor assay to investigate the role of the conserved disulfide bond in stabilizing the structure of GEs (Table 3). Melting curves can be found in supplementary Fig. 2. *Pr*CE15 displayed a multiphasic unfolding curve however, upon reduction of the disulfide bond, *Pr*CE15 unfolding was monophasic with the higher T_*m*_ unfolding event no longer detectable. Similarly, the addition of a reducing agent lowered the T_*m*_ of *Fs*CE15 by 11.5 °C. These results demonstrates the important role that the disulfide bond in the active site of CE15-B GEs plays in stabilizing tertiary structure. In contrast, the addition of reducing agent to *Rf*CE15 did not alter its thermal stability, an observation consistent with the lack of disulfide bonds. The influence of ionic strength on protein stability was also examined, but the thermal stability of the GEs was not altered by the addition of up to 500 mM NaCl.

**Table 3:** Mean unfolding temperature (T_*m*_) of *Fs*CE15, *Pr*CE15, and *Rf*CE15 in the presence and absence of reducing agent (TCEP) and at increasing ionic strength. The standard error of the mean T_m_ calculated in duplicate experiments is shown

	Control buffer*	10 mM TCEP	100mM NaCl	500 mM NaCl
*Pr*CE15	44.5 ± 0.6/59.9 ± 0.3	49.5 ± 0.47	41.2 ± 0.3/59.8 ± 0.4	42.5 ± 0.3/59.4 ± 0.2
*Rf*CE15	44.4 ± 0.04	46.3 ± 0.18	44.5 ± 0.19	44.2 ± 0.04
*Fs*CE15	48.7 ± 0.2	37.2 ± 0.37	47.9 ± 0.23	47.5 ± 0.1

* 3-component buffer system: 25 mM acetic acid, 25 mM MES, 50 mM Tris-HCl pH 7.0

## Discussion

*Glucuronoyl Esterases in the Rumen Share Characteristics of Both Prokaryotic and Eukaryotic Enzymes:* GEs can be broadly defined in terms of “bacterial“ and “fungal“ origin however, our characterization of these enzymes indicates that this clear differentiation for members of the rumen microbiome is questionable. Sequence based phylogenetic analysis of the carbohydrate esterase family 15 CAZy family has previously found that bacterial GEs display more sequence diversity than fungal GEs [8, 15]; a conclusion supported by our analysis (Fig. 1). When the structural features of GEs are considered alongside sequence, although the GEs from *F. succinogenes* and *R. flavefaciens* are bacterial in origin, they both exhibit structural features that are typically observed in fungal GEs. This observation brings up the question as to whether the observed similarity between bacterial and fungal GEs in the rumen arose as a result of an inter-kingdom horizontal gene transfer (HGT) event(s). AGF diverged from a common ancestor ~ 66 Mya, coinciding with the emergence of terrestrial grasses [49]. At this time, there was a shift in the diets of terrestrial mammals towards herbivory and the emergence of ruminants ~ 50 Mya [1]. The intense selective pressures, and unique physicochemical properties that are found in the rumen likely influenced the evolution of AGF.

Neocallimastigomycota are known to possess some of the highest densities of carbohydrate-active enzymes CAZymes of any characterized microbes, with many of themse CAZymes showing low homology to characterized enzymes. AGF also tend to have larger genomes than aerobic fungi due to high levels of gene duplication. This is particularly true of CAZymes, with 25–40% of these genes in AGF having undergone duplication [50–52]. This is clearly evident in GEs, with AGF possessing an average of four GE containing genes in there genomes. It has been hypothesized that microbes encode multiple GE orthologues as a result of each one having distinct biological roles [15]. The rumen microbiome is inhabited by multiple species of AGF and the multiplicity in GE genes observed here may have evolved to enable AGF to effectively complement the enzyme activities of the rumen bacteria in the presence of diverse substrates. This is known to occur within members of Neocallimastigomycota which have evolved mechanisms to target components of the plant cell wall distinct from those of bacteria, thereby facilitating their co-existence in the highly competitive rumen environment [53, 54]. It is possible that HGT between rumen bacteria and AGF also contributed to the expansion of the number of GEs found in the Neocallimastigomycota genome. There is evidence of extensive HGT between CAZymes from rumen bacteria to AGF with over 50% of the carbohydrate esterase family 15 genes in AGF showing evidence that they originated in rumen bacteria [52]. Interestingly, HGT events between Fibrobacterota and AGF were one of the most commonly observed events by Murphey at al. [52]. This observation is supported by our phylogenetic analysis showing that *Fs*CE15 clustered closely with Neocallimastigomycota carbohydrate esterase family 15 esterases (Fig. 1). *Fs*CE15 also possesses structural features that are typically observed in fungal GEs including: a shallow active site lacking any of the insertions in the Reg1-Reg3 regions, catalytic residues that adopt a canonical CE15-A active site conformation, and a conserved disulfide bond in the active site. Although we cannot say which organism this gene may have originated in, the similarity between *Fs*CE15 and *Pr*CE15 may have resulted due to an HGT event that occurred between *F. succinogenes* and *P. rhizinflata* at some point in the evolutionary past. *Fs*CE15 is therefore unique amongst bacterial GEs characterized to date and may represent an intermediate between fungal and bacterial GEs.

*GEs Play a Role in the Metabolism of Lignocellulose by Rumen Microbes But Their Physiological Substrate Is Unclear:* The biochemical properties and kinetic parameters of *Fs*CE15, *Pr*CE15, and *Rf*CE15 are consistent with previously characterized GEs. All three enzymes showed specificity for BnzGlcA but, displayed low affinity and poor catalytic efficiency against synthetic substrates. It is likely that *Fs*CE15, *Pr*CE15, and *Rf*CE15 would show higher levels of catalytic activity against physiological substrates that they target in vivo [13, 15]. Unfortunately, the role of glucuronic acid – lignin cross-linkages in the structural integrity of the cell wall of the crops and grasses typically consumed by ruminants is unclear. Recently, it has been suggested that GEs not only target the LCC-hemicellulose complex but may also play a role in breaking down pectin-lignin LCCs [55]. It is known that genes encoding GEs are expressed during the hydrolysis of lignocellulosic substrates in the rumen [56]. Furthermore, transcriptomic analysis of lignocellulose digestion by *P. rhizinflata* found that genes encoding GEs are expressed during growth on corn stover, barley straw, and alfalfa; suggesting that these enzymes play a role in the utilization of lignocellulose by the rumen microbiome [53, 56].

Although it has been shown that supplementing enzyme cocktails with GEs can result in improved saccharification [15], no improvements in glucose or xylose release from corn stover or barley straw was observed by the GEs characterized in this work (data not shown). Due to the challenges associated with isolating natural LCC substrates, almost all GEs characterized to date have relied on the use of model substrates. Further research is needed to identify potential LCC substrates in ruminant feeds which would enable GEs from rumen microbes to be characterized using physiologically relevant substrates.

*The Structural Properties of Rumen GEs Are Consistent with Previously Characterized Members of the Carbohydrate Esterase 15 CAZy Family:* Consistent with other GEs, *Fs*CE15, *Pr*CE15, and *Rf*CE15 adopt a canonical α/β hydrolase fold (Fig. 2), and possess a structurally conserved Ser-His-Glu/Asp catalytic triad (Figs. 3 and 4). Structural variation between *Fs*CE15, *Pr*CE15, and *Rf*CE15 was limited to surface loops surrounding the active site, and these differences were correlated with the presence or absence of a disulfide bond in the active site. Although the position of the catalytic nucleophile and general base is highly conserved in GEs, the general acid has been observed at 2 distinct locations which has led to GEs being classified as possessing a canonical (CE15-B) or non-canonical (CE15-A) catalytic triad [24]. The canonical configuration of the catalytic residues has a Glu located on a loop after β7, whereas the non-canonical configuration has a Glu/Asp located on a flexible loop after β8. There are also examples of GEs that have an acidic residue at both sites, providing functional redundancy in these enzymes [22]. All structurally characterized GEs within the CE15-B subfamily are bacterial in origin and lack the disulfide bond that anchors the general base into position to enable optimal hydrogen bonding with the nucleophilic serine (Fig. 3). In contrast, all of the previously structurally characterized GEs within the CE15-A subfamily posses this covalent bond and originate from fungi. The presence of this disulphide bond also provides stability to GEs, demonstrated by the structural destabilization of the protein structure and lowering of its melting temperature upon reduction of this covalent bond (Table 3). The physiological temperature of the rumen is 39 °C, so it does not seem likely that high thermal stability would be important for these enzymes in vivo. This feature may also contribute to the stability of these enzymes in other ways including protease stability however, this has not been confirmed in vitro.

In the absence of the disulfide bond within the non-canonical CE15-B GE subfamily, extensive networks of Van der Waals interactions and H-bonding likely play a similar stabilizing role. The non-covalent nature of the interactions with the general base loop in CE15-B subfamily enzymes would provide stability to the general base loop, while still providing conformational flexibility. It is likely that the lack of a covalent anchor in the active site provides these enzymes with the structural plasticity required to bring the non-canonical general acid within hydrogen bonding distance of the general base. This conformational flexibility has also been suggested to play a role in the substrate specificity of GEs [15, 16, 21].

CAZymes commonly adopt multi-domain structures composed of complementary enzyme activities that function synergistically to hydrolyze complex polysaccharides. We were unable to express a multidomain construct of CesA, however a search of the AlphaFold database identified a model of the full length *R. flavefaciens* CesA (AlphaFold model ID: Q9RLB8) [57]. Interestingly, AlphaFold predicts that the active sites of the N-terminal carbohydrate active enzyme 3 family, and C-terminal carbohydrate active enzyme 15 family, domains could adopt a low energy conformation in which the GE and acetylxylan esterase catalytic sites are oriented towards each other (supplementary Fig. 3). This tertiary arrangement could facilitate the simultaneous cleavage of an ester bond between 4-*O*-methyl-d-glucuronyl in xylan and lignin alcohol groups in LCCs by the GE domain, and deacetylation of the xylan backbone of D-glucuronylxylan by the acetylxylan esterase domain. We hypothesize that CesA may be an interesting model system to study enzyme cooperativity in multi-domain CAZymes [47, 48]. Due to the theoretical nature of the CesA AlphaFold model, experimental validation is required to draw any such conclusions.

## Conclusion

We have experimentally determined the 3-dimensional structures and biochemical properties of three glucuronoyl esterases; two encoded by rumen prokaryotes and one encoded by a rumen fungus. Furthermore, we have examined the diversity of the carbohydrate esterase family 15 esterase family found in the rumen. Phylogenetic and structural analysis of rumen GEs lead to the hypothesis that interkingdom HGT events may have contributed to the diversity of GEs in the rumen, and that *Fs*CE15 may represent an intermediate between fungal and bacterial GEs. This examination of the GE active site structures highlights the importance of structural plasticity in carbohydrate esterase family 15 esterases with a non-canonical active site conformation, and suggests that this flexibility may play a role in defining GE substrate specificity. These data expand our current understanding of the structure-function relationship in glucuronoyl esterases and illuminates the evolutionary dynamics that contribute to enzyme diversity in the rumen microbiome.

### Electronic Supplementary Material

Below is the link to the electronic supplementary material.

Supplementary Material 1

Supplementary Material 2

## Data Availability

Structures of FsCE15, PrCE15, and RfCE15 have been deposited in the PDB under the accession codes 8TRU, 8TRX, and 8TSE, respectively.
