# Supplementary material for: Structural, Biochemical, and Phylogenetic Analysis of Bacterial and Fungal Carbohydrate Esterase Family 15 Glucuronoyl Esterases in the Rumen
Source: Protein J. 2024 Aug 17;43(4):910–22. doi: 10.1007/s10930-024-10221-0 (PMC11345330; doi:10.1007/s10930-024-10221-0)

**Supplementary Figure 1**: pH profiles for the hydrolysis of Benzyl D-glucuronate by *Pr*CE15(yellow), *Rf*CE15(Blue) and *Fs*CE15(green). Relative specific activity as compared to the activity at the optimal pH of each enzyme is shown.

**Supplementary Figure 2:** Thermofluor Melting curves for (A) *Pr*CE15, (B) *Rf*CE15 and (C) *Fs*CE15 examining the impact of a reducing agent and increasing ionic strength on protein stability.

A)


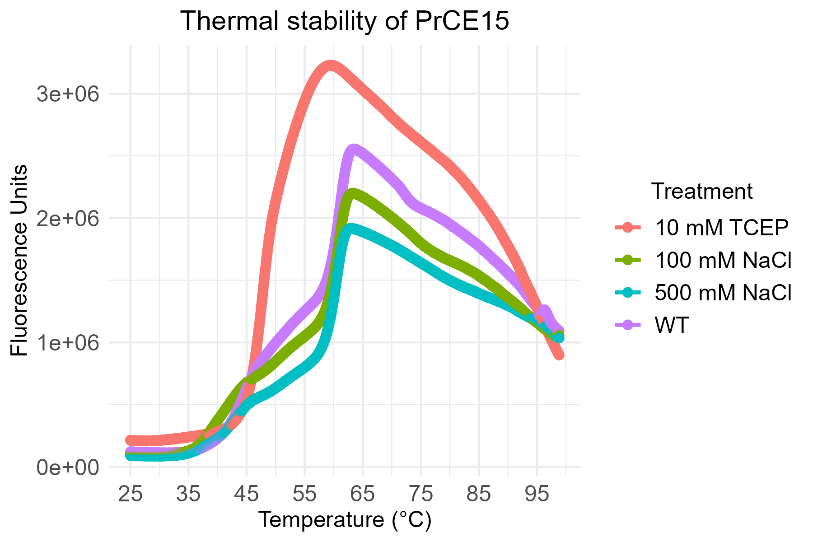


B)


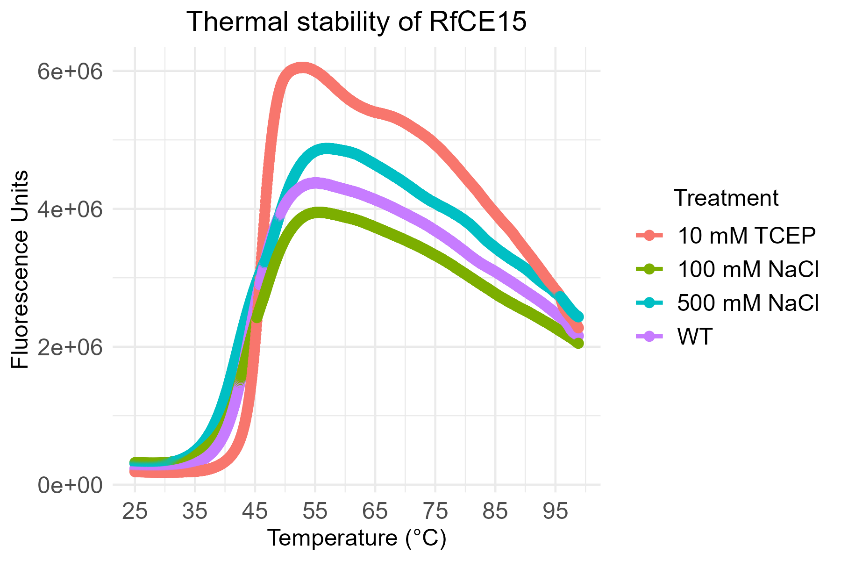


C)


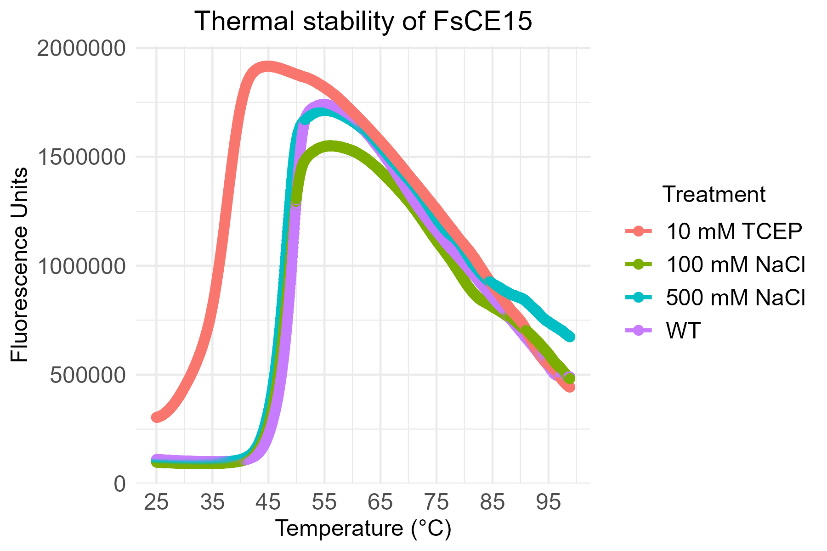


**Supplementary Figure 3:** AlphaFold model of the structure of full length CesA from Ruminococcus flavefaciens. The N-terminal carbohydrate esterase family 3 domain (wheat), dockerin domain (cyan) and carbohydrate esterase family 15 domain (light blue) are shown. Catalytic residues of both RfCE3 and RfCE15 domains are shown as spheres.


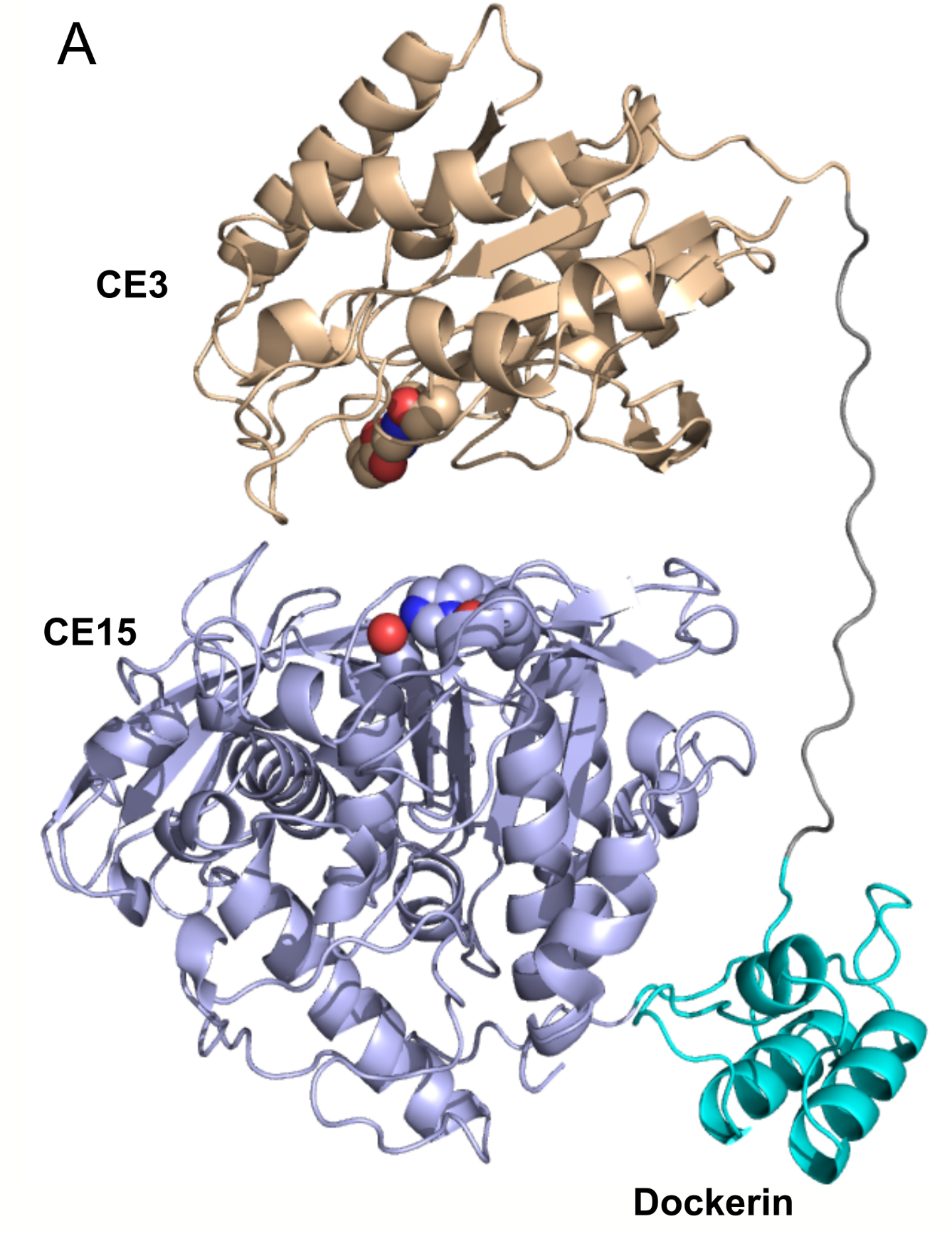

Supplement: Supplementary file 2 — Supplementary Material 2 [file 10930_2024_10221_MOESM2_ESM.docx]
